# An ATM/Chk2-mediated DNA damage responsive signaling pathway suppresses Epstein-Barr virus transformation of primary human B cells

**DOI:** 10.1016/j.chom.2010.11.004

**Published:** 2010-12-16

**Authors:** Pavel A. Nikitin, Christopher M. Yan, Eleonora Forte, Alessio Bocedi, Jason P. Tourigny, Robert E. White, Martin J. Allday, Amee Patel, Sandeep S. Dave, William Kim, Katherine Hu, Jing Guo, David Tainter, Elena Rusyn, Micah A. Luftig

**Affiliations:** 2 Department of Molecular Genetics and Microbiology, Center for Virology, Duke University School of Medicine, Durham, NC, 27712 USA; 3 Department of Virology, Faculty of Medicine, Imperial College London, Norfolk Place, London W2 1PG, United Kingdom; 4 Duke Institute for Genome Sciences and Policy, Duke University, Durham, NC, 27712 USA

## Abstract

Epstein-Barr virus (EBV), an oncogenic herpesvirus that causes human malignancies, infects and immortalizes primary human B cells *in vitro* into indefinitely proliferating lymphoblastoid cell lines, which represent a model for EBV-induced tumorigenesis. The immortalization efficiency is very low suggesting that an innate tumor suppressor mechanism is operative. We identify the DNA damage response (DDR) as a major component of the underlying tumor suppressor mechanism. EBV-induced DDR activation was not due to lytic viral replication nor did the DDR marks co-localize with latent episomes. Rather, a transient period of EBV-induced hyper-proliferation correlated with DDR activation. Inhibition of the DDR kinases ATM and Chk2 markedly increased transformation efficiency of primary B cells. Further, the viral latent oncoproteins EBNA3C was required to attenuate the EBV-induced DNA damage response We propose that heightened oncogenic activity in early cell divisions activates a growth-suppressive DDR which is attenuated by viral latency products to induce cell immortalization.

## INTRODUCTION

Epstein-Barr virus (EBV) is an oncogenic herpesvirus causally implicated in several malignancies including African endemic Burkitt's lymphoma, post-transplant lymphoproliferative disease, nasopharyngeal carcinoma, and HIV-associated lymphomas (Kieff and Rickinson, 2006). EBV infection *in vitro* drives primary human B cells into indefinitely proliferating lymphoblastoid cell lines (LCLs) providing a model for tumorigenesis. This process of growth transformation depends on a subset of viral latent oncoproteins and non-coding RNAs collectively termed ‘latency III’. The proteins expressed include the Epstein-Barr nuclear antigens, EBNA1, 2, 3A, 3B, 3C, and LP as well as three latent membrane proteins, LMP1, 2A, and 2B. EBNA-LP and EBNA2 are the first viral proteins expressed following primary B cell infection (Alfieri et al., 1991) and up-regulate cellular genes inducing a transition of resting B cells into the cell cycle (Sinclair et al., 1994; Wang et al., 1991). EBNA2 also induces expression of the remaining EBNA proteins (Zimber-Strobl et al., 1993) and subsequently the viral latent membrane proteins, LMP1 and LMP2A/2B (Wang et al., 1990).

While the initial burst of viral and cellular gene expression leads to the proliferation of infected cells *in vitro*, only a small percentage of infected cells become indefinitely proliferating lymphoblasts (Henderson et al., 1977; Sugden and Mark, 1977). The study of EBV-induced innate tumor suppressor pathways has been limited. EBV infection of primary B cells induces the p53 protein concomitant with EBNA-LP expression early after infection (Szekely et al., 1995). However, it remains unclear whether this innate response to EBV-induced proliferation has any long-term functional consequence or what pathways activate p53.

Innate tumor suppressor responses have been better characterized in other systems. The DNA damage response (DDR) has recently been appreciated as an important tumor suppressor pathway *in vitro* and *in vivo* (Bartkova et al., 2005; Gorgoulis et al., 2005). The DDR is triggered by aberrant replication structures generated by activated oncogenes attempting to constitutively fire new origins and inappropriately enter S phase (Halazonetis et al., 2008). The DDR limits aberrant proliferation by mediating oncogene-induced senescence and apoptosis (Bartkova et al., 2006; Di Micco et al., 2006). Signaling downstream of oncogenic stress involves activation of the single-stranded DNA-dependent ATR pathway and the double-stranded break-induced ATM pathway. These DDR kinases relay downstream signals to critical repair factors and other checkpoint kinases including Chk1 and Chk2 with extensive cross-talk ultimately resulting in suppression of oncogene-induced proliferation (Halazonetis et al., 2008; Stiff et al., 2006). Genetic experiments have identified critical roles for ATM and Chk2 in mediating oncogene-induced senescence and tumor suppression (Bartkova et al., 2006; Pusapati et al., 2006; Stracker et al., 2008). Given these observations and the low efficiency of EBV transformation, the intriguing question remains as to whether the host DNA damage response senses EBV-induced oncogenic stress and, importantly, if this is responsible for the block to long-term outgrowth of the majority of infected cells.

## RESULTS

### Epstein-Barr virus infection of primary B cells activates a cellular DNA damage response

**Figure 1:**
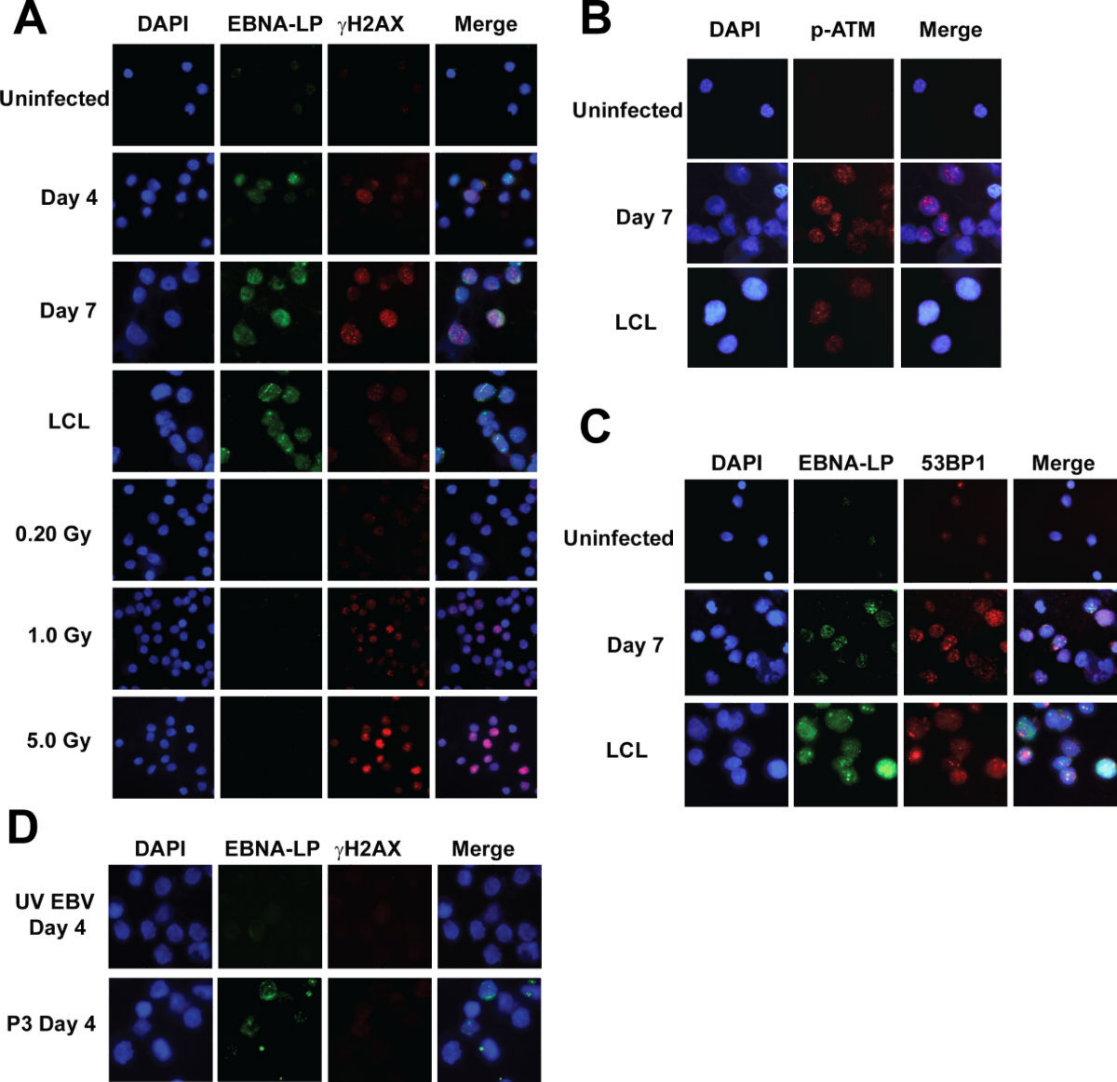
EBV induced a DNA damage response in primary B cells **(A)** Indirect immunofluorescence (IF) images of EBNA-LP (green) and γ-H2AX (red) in uninfected B cells, B cells 4 and 7 days after infection with EBV B95-8 (MOI ~5), the recently derived LCL EF3D, and uninfected γ-irradiated B cells (0.2, 1, and 5 Gy, 1h). DNA is stained with DAPI. These images are representative of infections in five different normal donors. **(B)** Ser1981 phosphorylated ATM (pATM, red) in uninfected B cells and B cells 7 days after EBV B95-8 infection. EBNA-LP or other EBV latent antigen staining was not possible in these samples due to antibody source, however we know that ~50% of these infected cells are EBNA-LP-positive. **(C)** EBNA-LP (green) and 53BP1 (red) in uninfected B cells and B cells 7 days after infection. **(D)** EBNA-LP (green) and γ-H2AX (red) in B cells 4 days after infection with UV-inactivated EBV B95-8 (UV EBV) or the non-transforming EBV strain P3HR1 (P3).

We first sought to determine whether EBV infection of primary B cells might drive an oncogenic stress leading to the activation of the DNA damage response. Purified CD19+ B cells were infected with the prototypical transforming EBV strain B95-8 at a multiplicity of infection (MOI) of ~5. Nearly all cells were EBV genome positive as determined by fluorescence *in situ* hybridization (FISH) (Fig. S1A). Infected cells were initially assayed for the expression of the earliest viral latency gene product, EBNA-LP (LP), and the DNA damage marker, γ-H2AX, at different times post infection. γ-H2AX activation was not evident prior to 4 days post infection, was robust from 4 to 7 days post infection, and declined after 7 days to the low levels observed in LCLs (Fig. 1A and data not shown). Approximately 60% of the infected cells were γ-H2AX positive at 7 days post infection. Corroborating our findings of γ-H2AX activation, EBV infection induced additional hallmarks of the DDR including auto-phosphorylation of the H2AX kinase ATM (pATM Ser1981), and punctate localization of the damage adaptor 53BP1 (Fig. 1B and 1C).

EBV gene expression was important for virus-induced DDR activation. Cells infected with UV-inactivated B95-8 virus did not show γ-H2AX staining at any point within the first week after infection (Fig. 1D and data not shown). Importantly, UV-inactivated EBV B95-8 genomes reached the nucleus and these infections induced interferon-responsive genes (Fig. S1A and B). EBNA2 and latency III gene expression was specifically necessary to induce the DDR as B lymphocytes infected with the EBNA2 deleted, transformation-incompetent P3HR1 strain of EBV did not contain γ-H2AX foci (Fig. 1D) despite similar levels of infection compared to B95-8 (Fig. S1A-C). These data collectively demonstrate that EBV latent gene expression rather than simply virion binding or nucleic acid deposition into the nucleus was required to induce γ-H2AX activation.

### The EBV-induced DNA damage response in primary B cell infection is not associated with viral episomes or lytic replication

We reasoned that either viral or cellular DNA may activate the DNA damage response. Since evidence in the literature suggested that either viral lytic DNA replication (Kudoh et al., 2005) or latent viral episome replication (Dheekollu et al., 2007) may be capable of inducing a DDR, we first assayed viral DNA as a possible source of the damage. Incoming linear viral DNA was not the source of the damage since UV-irradiated and EBNA2-deleted P3HR1 virus infections did not induce the DDR (Fig. 1). We next used a FISH based assay to assess the possible role of lytic DNA replication. The B95-8 Z-HT cell line was used as a positive control where lytic EBV DNA was recognized as a brightly staining FISH signal rather than the punctate foci of episomal genomes (Fig. S1D). Less than 1% of EBV-infected cells contained evidence of lytic viral DNA 5 days post infection, while approximately 1-5% of infected cells were spontaneously undergoing lytic replication by 14 days similar to that found in LCLs (Fig. S1E and (Kieff and Rickinson, 2006)). Since far greater than 1% of EBV-infected cells were γ-H2AX positive early after infection, we conclude that viral lytic DNA replication is not responsible for DDR activation.

Next we assessed the possibility that latent viral episomes activate the DNA damage response. The mean episome number per cell as assessed by FISH did not increase during the period when γ-H2AX activity was high early after infection (Fig. S1F). Furthermore, we failed to observe significant co-localization of EBV episomes with γ-H2AX foci in these cells (Fig. S1G). In fact, the number of γ-H2AX foci per cell was consistently much greater than the number of EBV genomes (Fig. S1G). Therefore, our data collectively suggest that the observed EBV-induced DDR is not activated by viral DNA.

### The EBV-induced DNA damage response is associated with a transient period of hyper-proliferation

**Figure 2:**
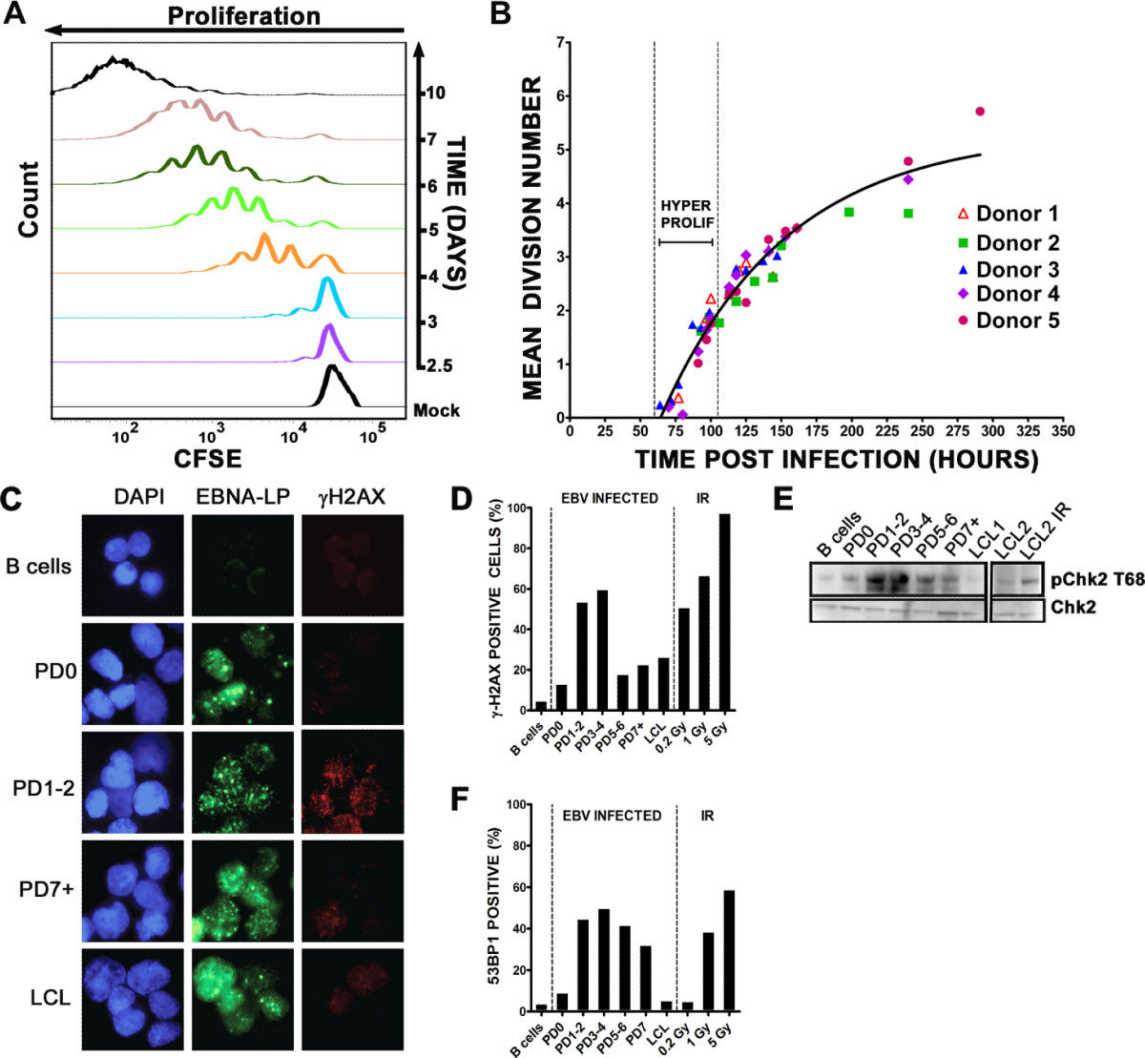
EBV induced a period of hyper-proliferative early after infection that was associated with activation of the DNA damage response **(A)** Histograms show CD19+ B cell division as measured by CFSE staining at different days after EBV infection. Mock, mock infected cells. **(B)** The mean division number based on precursor cohort analysis for EBV-infected B cells is plotted at different times post infection. Vertical dashed lines estimate the hyper-proliferation period. Data are presented from 5 normal donors. **(C)** IF of γ-H2AX (red) and EBNA-LP (green) of uninfected cells, infected cells that have yet to divide (PD0), infected cells after 1 or 2 divisions (PD1-2), or 7 or more divisions (PD7+) and LCLs. DNA is stained with DAPI. **(D)** The percentage of EBNA-LP positive cells with γ-H2AX signal >5X over background is graphed from uninfected B cells, sorted PDs, and LCLs. Uninfected B cells following 0.2, 1, and 5 Gy (1hr) γ-irradiation are also shown as a positive control. These data are representative of similar experiments from three independent normal donors. **(E)** Immunoblot of p-Chk2 Thr68 and Chk2 in sorted cells as in (**D**) including an LCL following 5 Gy γ-irradiation (1hr). (**F**) The percentage of EBNA-LP positive cells containing 4 or more 53BP1 foci per cell in sorted populations as in (**D**) are shown along with uninfected irradiated B cell controls. PD3-4 contained significantly more 53BP1 foci per cell than uninfected B cells (p<0.0001), PD0 (p<0.0001), PD7 (p<0.01), and LCL (p<0.0001).

We next focused our studies on changes in cellular DNA that may induce a DDR. The period of time post infection when the DDR was active correlates with the initiation of B cell proliferation (Kieff and Rickinson, 2006). Analysis of CD19+ B cells using the proliferation-tracking dye CFSE at different days after infection indicated that i) proliferating cells appeared at day 3 (Fig. 2A), ii) between days 3 and 4 there were always cells that had divided more than once or even twice in 24h, and iii) at later days post infection cells appeared to proliferate at a slower rate as judged by the less pronounced shift of the CFSE profile to the left.

A more rigorous kinetic analysis of EBV-induced B cell expansion highlighted the biphasic nature of the proliferation rate (Fig. 2B). Infected CD19+ B cell CFSE profiles from five normal donors were analyzed at time points prior to and during the first seven cell divisions. The mean division number (MDN) at each time point was determined by fitting the precursor-normalized number of cells in each division to a Gaussian distribution (Fig. S2A and (Hawkins et al., 2007)). The slope of the function relating MDN to time post infection inversely correlates with the proliferation rate. Consistent with the data in Fig. 2A, we observed that EBV induced an early phase of hyper-proliferation that was attenuated over time (Fig. 2B). The proliferation rate of initially proliferating cells was approximately once per 8-12h while later cycles were ~24-30h similar to the ~24-28h rate of LCLs. These findings were corroborated by cell sorting experiments where cells from earlier divisions proliferated more quickly than those in later divisions (Fig. S2B). Thus, EBV-mediated B cell expansion proceeds through an initial period of hyper-proliferation followed by slower cell divisions typical of emergent LCLs.

We next asked whether the DNA damage response was activated specifically during the hyper-proliferative divisions independent of time post infection. EBV-infected B cells sorted based on population doubling (PD) were subjected to immunofluorescence for EBNA-LP and γ-H2AX (Fig. 2C). Sorted cells were >85% EBNA-LP positive in cells not yet dividing (PD0) and >95% EBNA-LP positive in all later PDs. We observed a robust increase in LP^+^/γ-H2AX^+^ cells during the early PDs (1-2 and 3-4) relative to uninfected cells or infected cells not yet proliferating (PD0) (Fig. 2C and 2D). Importantly, this response was attenuated through later PDs and in LCLs. Moreover, γ-H2AX intensity per cell was significantly higher in PD3-4 than PD0 (p<0.0001) and LCL (p<0.0001). We also observed a transient activation and attenuation of the ATM-specific phosphorylation of Chk2 on Thr68 (Fig. 2E) as well as accumulation of 53BP1 into DDR foci (Fig. 2F). These data strongly support the notion that the EBV-induced DDR is caused by an early period of hyper-proliferation and is attenuated during LCL outgrowth.

### Proliferation and DNA damage responsive genes are highly induced early after EBV infection, then attenuated during LCL outgrowth

**Figure 3:**
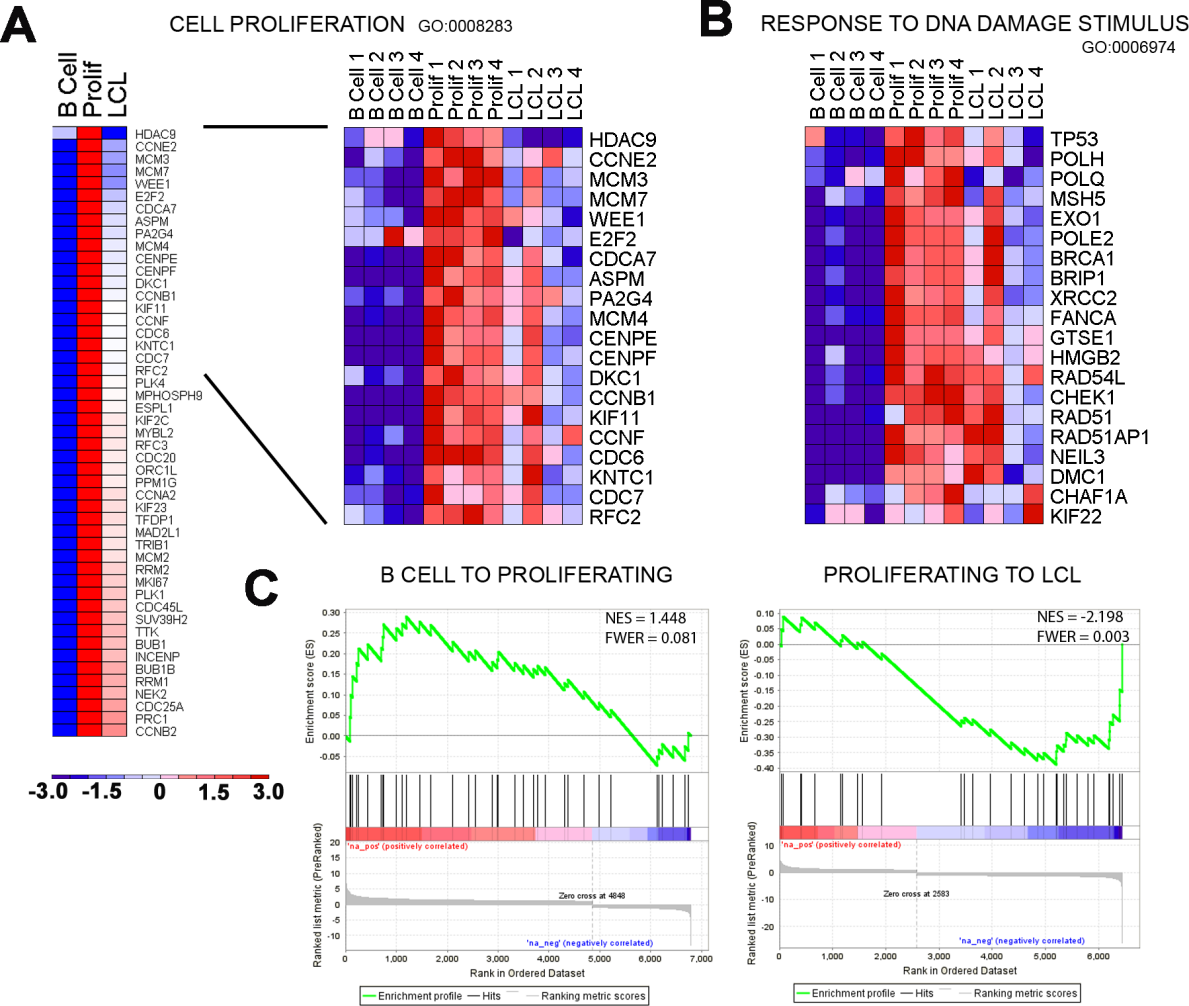
Transcriptional changes correlate with an EBV-induced early period of hyper-proliferation and DNA damage response followed by attenuation upon LCL outgrowth (**A**) Heatmap of average expression data across four normal donors for the gene ontology (GO) category “Cell Proliferation” in uninfected resting B cells (**B cell**), EBV-infected early proliferating B cells (**Prolif**), and monoclonal LCLs (**LCL**). The genes presented were derived from GATHER analysis of all genes with significant expression changes (2-way ANOVA, p<0.01) where the expression level increased from **B cell** to **Prolif** at least 1.5-fold and decreased from **Prolif** to **LCL** at least 1.2-fold (**left**). Heatmap of individual samples of top 20 “Cell Proliferation” genes (**right**). (**B**) Heatmap of “Response to DNA Damage Stimulus” GO genes across individual samples. (**C**) Gene Set Enrichment Analysis (GSEA) of known DNA damage induced ATM and p53-depending genes in the context of **B-Prolif-LCL** expression data. The reference list of ATM/p53 target genes was derived from Clusters 2 and 3 of (Elkon et al., 2005) and compared with a pre-ranked list (by fold) of global average gene expression changes from **B cell** to **Prolif** (**left**) and **Prolif** to **LCL** (**right**). Statistical scores are inset into the top right of analysis images (NES: Normalized enrichment score and FWER: Familywise error rate).

Our cell-based findings were corroborated by mRNA microarray studies of i) uninfected B cells, ii) EBV-infected early proliferating cells (Prolif), and iii) monoclonal LCLs from four normal donors (Fig. 3). We first asked in an unbiased manner which genes were significantly changed upon proliferation and then, subsequently, during LCL outgrowth (2-way ANOVA, p<0.01). As expected, the most enriched gene ontology (GO) category for genes induced from resting B cells to EBV-infected, proliferating B cells was ‘Cell Proliferation’ (Fig. 3A; GO:0008283, Bayes factor: 51, p<0.0001 (Chang and Nevins, 2006)). Genes associated with the ‘Response to DNA Damage Stimulus’ were also highly induced (Fig. 3B; GO:0006974, Bayes factor: 17, p<0.0001). Notably, we observed that the majority of genes involved in cell proliferation and the DNA damage response were consistently repressed as cells transitioned from early proliferating to established LCLs (Fig. 3A, Cell Proliferation, Bayes factor: 63, p<0.0001 and Fig. 3B, Response to DNA Damage Stimulus, Bayes factor: 22, p<0.0001). Consistently, the expression of genes in an independently derived set of DNA damage responsive and ATM-dependent p53 targets (Elkon et al., 2005) was also increased in early proliferating cells and subsequently attenuated during LCL outgrowth (Fig. 3C and S3). Collectively, these global gene expression analyses corroborate our findings of a period of hyper-proliferation and activation of an ATM-dependent DNA damage response early after infection that is attenuated during LCL outgrowth.

### The EBV-induced hyper-proliferation associated DNA damage response is growth suppressive

**Figure 4:**
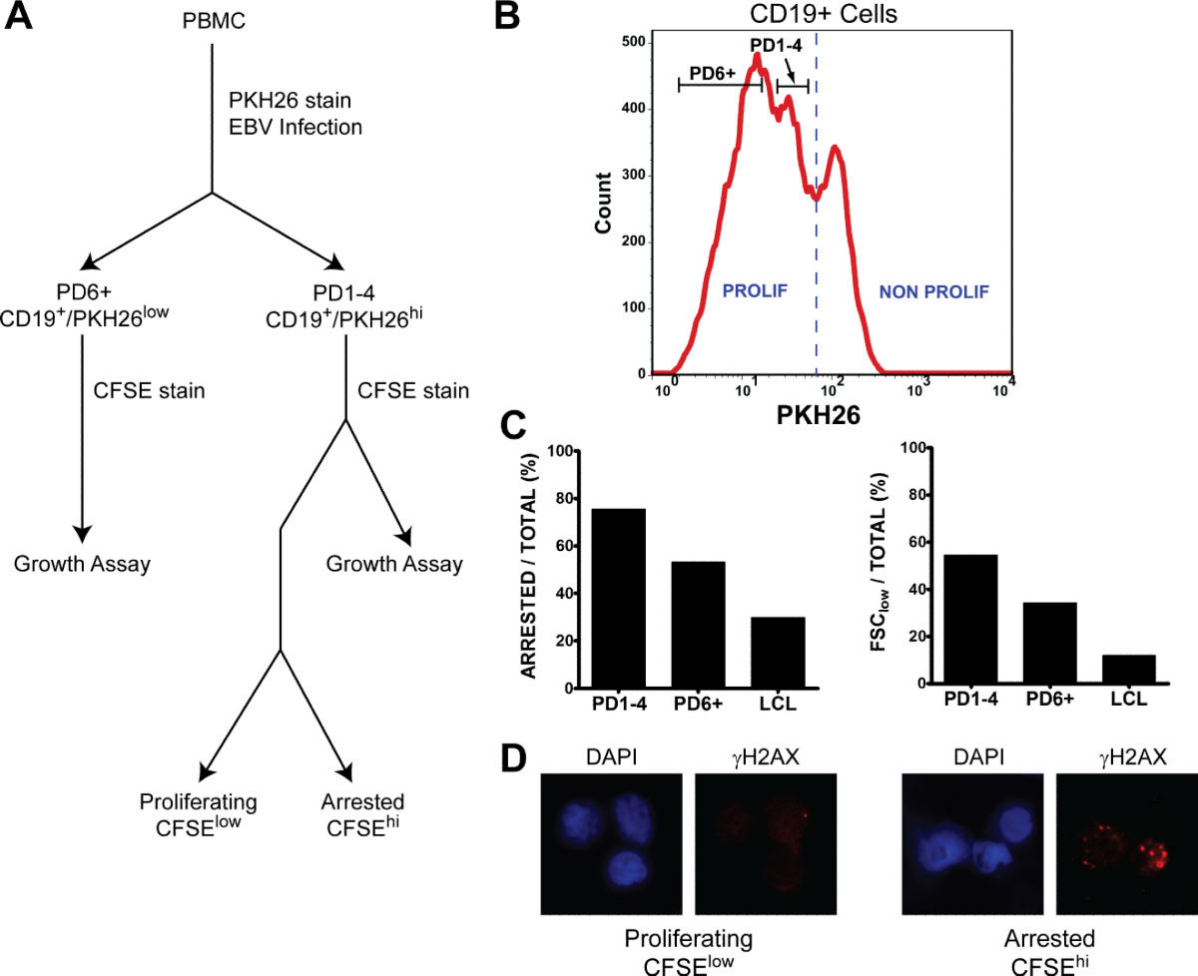
Growth suppression and DNA damage enrichment in early cell divisions (**A**) A flowchart shows the separation of arrested and proliferating EBV-infected CD19+ PBMC used for IF and FACS. PBMC were first infected with EBV and labeled with the red fluorescent dye PKH26. (**B**) Then, 8 days post infection, proliferating CD19+ B cells were sorted for PD1-4 and PD6+ based on PKH26 intensity, labeled with CFSE, and cultured for two days. (**C**) Sorted cells were then analyzed in a FACS-based growth assay where cells in the CFSE^low^ population were considered proliferating and cells in the CFSE^hi^ population were considered arrested. Forward scatter (FSC) low reflects dying cells. Results are representative of three normal donors. (**D**) PKH26^low^ (PD1-4) cells were subsequently labeled with CFSE as above, sorted after 48h in culture into CFSE^hi^ (arrested) and CFSE^low^ (proliferating) populations, and analyzed by IF for γ-H2AX (red).

To further analyze the consequences of the activated DDR in early rapidly proliferating cells, we designed a sorting strategy to assess the relative growth potential and DDR activation in cells derived from early or late divisions after infection (Fig. 4A). We initially stained cells with the proliferation tracking dye PKH26 and sorted cells after infection for PD1-4 and PD6+ populations (Fig. 4B). Subsequent staining with CFSE enabled the analysis of proliferation from these populations. Supporting our hypothesis, the cells in early hyper-proliferating divisions (PD1-4) were, in fact, more prone to growth arrest and cell death than those in later divisions (PD6+) and LCLs (Fig. 4C). Consistently, arrested PD1-4 cells displayed significantly more intense γ-H2AX staining than their proliferating counterparts (Fig. 4D).

### ATM and Chk2 kinases suppress EBV-mediated transformation and initial B cell proliferation

**Figure 5:**
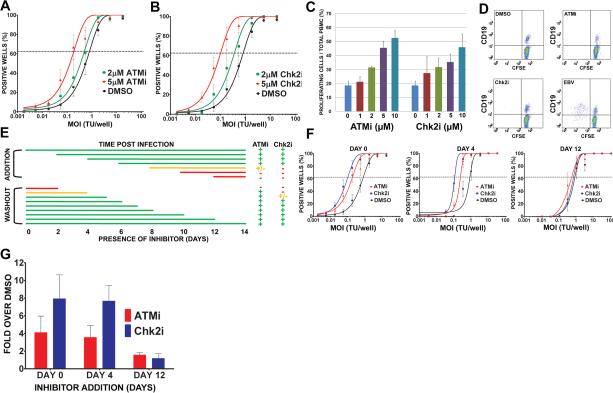
Inhibition of ATM and Chk2 kinases increased EBV transformation efficiency and proliferation of B cells during a critical period 4-8 days post infection **(A)** Quantification of EBV-induced B cell outgrowth following PBMC infection in the presence of 0.1% DMSO (black), 2 μM ATMi (green), or 5 μM ATMi (red). The percentages of wells positive for LCLs at five weeks post infection are plotted relative to the transforming units (TU) of B95-8 virus per well. Results shown are the average of experiments with at least four independent normal donors. Error bars represent standard error of the mean (SEM). **(B)** Similar experiments were performed using DMSO (black), 2 μM Chk2i (green), or 5 μM Chk2i (red). **(C)** CFSE-stained PBMC were infected with EBV in the presence of increasing amounts of ATMi or Chk2i (DMSO, 1 μM, 2 μM, 5 μM, and 10 μM). The percentage of CD19^+^/CFSE^low^ cells of total PBMCs at 14 days post infection are plotted. The data shown are the average values from two different donors +/-SEM. These data are representative of more than five independent experiments. **(D)** Dot plots show CFSE-and CD19-stained PBMC that were treated with DMSO, 5 μM ATMi, 5 μM Chk2i, or infected with EBV for six days. **(E)** This table summarizes when ATM and Chk2 suppressed EBV-mediated proliferation at different times following infection. CFSE-stained PBMC were infected with EBV at day 0. ATMi or Chk2i (5 μM) was added at different times after infection (top) or at day 0 and washed out at different times after infection (bottom). EBV-mediated B cell proliferation was detected by FACS at day 14 post infection using CD19-PE and CFSE as in (**C**). A more than two-fold increase in treated cells versus DMSO is represented by a green plus; a less than two-fold increase is represented by a yellow plus; and no increase is represented by a red dash. The lines indicate the period of incubation and are colored with the proliferation phenotype after ATMi and Chk2i treatment. Average values from two independent donors are shown. **(F)** EBV-induced outgrowth following PBMC infection was measured as in (**A**) in the presence of 5 μM Chk2i (blue), 5 μM ATMi (red), or DMSO (black) added at day 0, day 4 or day 12 after EBV infection. Results shown are the average of four independent normal donors +/-SEM. **(G)** Efficiency of EBV outgrowth from (**F**) was calculated and the average ratio of inhibitor-treated to DMSO-treated infections +/-SEM for four normal donors is plotted.

To determine if the activation of the DDR restricts EBV-mediated long-term outgrowth, we simultaneously infected peripheral blood mononuclear cells (PBMC) with EBV and treated them with an inhibitor of either ATM (ATMi (Hickson et al., 2004)) or its downstream effector kinase Chk2 (Chk2i (Arienti et al., 2005)); both are critical kinases in the DDR checkpoint responding to DNA double-stranded breaks and oncogenic stress. EBV-mediated B cell transformation efficiency increased in a dose-dependent manner in response to the inhibitors where 2 μM ATMi increased efficiency by approximately 2-fold and 5 μM ATMi by 6-fold over DMSO control treated cells (Fig. 5A). Similarly, Chk2 inhibition increased EBV transformation efficiency approximately 3-fold for 2 μM Chk2i and 9-fold for 5 μM Chk2i (Fig. 5B). Therefore, an ATM and Chk2 dependent DDR restricts EBV transformation.

We next assessed whether ATM and Chk2-mediated suppression of EBV transformation was due to limiting initial B cell proliferation. The continuous presence of either ATM or Chk2 inhibitor led to a dose dependent increase in B cell number at two weeks post infection (Fig. 5C). Importantly, ATM or Chk2 inhibitor did not induce B cell proliferation in the absence of EBV suggesting that these compounds act to alleviate a block to proliferation rather than stimulating B cells per se (Fig. 5D).

### ATM and Chk2 suppress B cell growth 4-8 days after EBV infection

Since the DDR peaked during the first week after infection, we assessed when ATM and Chk2 inhibition enhanced proliferation and transformation. To that end, PBMC infected with EBV were transiently exposed to ATMi and Chk2i from the start of infection or the compounds were added at different days post infection. EBV-induced B cell proliferation was most sensitive to the inhibitors between 4 and 8 days after infection when cells were present in the hyper-proliferative period (Fig. 5E). For example, when either inhibitor was added within the first 4 days of infection we observed as pronounced an effect on proliferation as if the inhibitor was added at day 0. However, if we added inhibitors after day 8, there was no effect on proliferation. Conversely, if the inhibitors were removed prior to 4 days after infection, then increased proliferation was not observed.

Similar results were obtained in long-term transformation assays. Addition of either compound within 4 days of infection increased transformation efficiency, while adding the compounds at 12 days post infection had little effect (Fig. 5F and 5G). The inhibitors also did not increase LCL growth rates at normal or limiting density (Fig. S4 and data not shown). Therefore, during a critical period approximately 4-8 days following infection, EBV induced an ATM and Chk2-dependent growth suppressive signaling pathway that limited initial B cell proliferation and, consequently, long-term outgrowth into lymphoblastoid cell lines.

### EBV latent gene expression changes and consequences in early infected cell divisions

**Figure 6:**
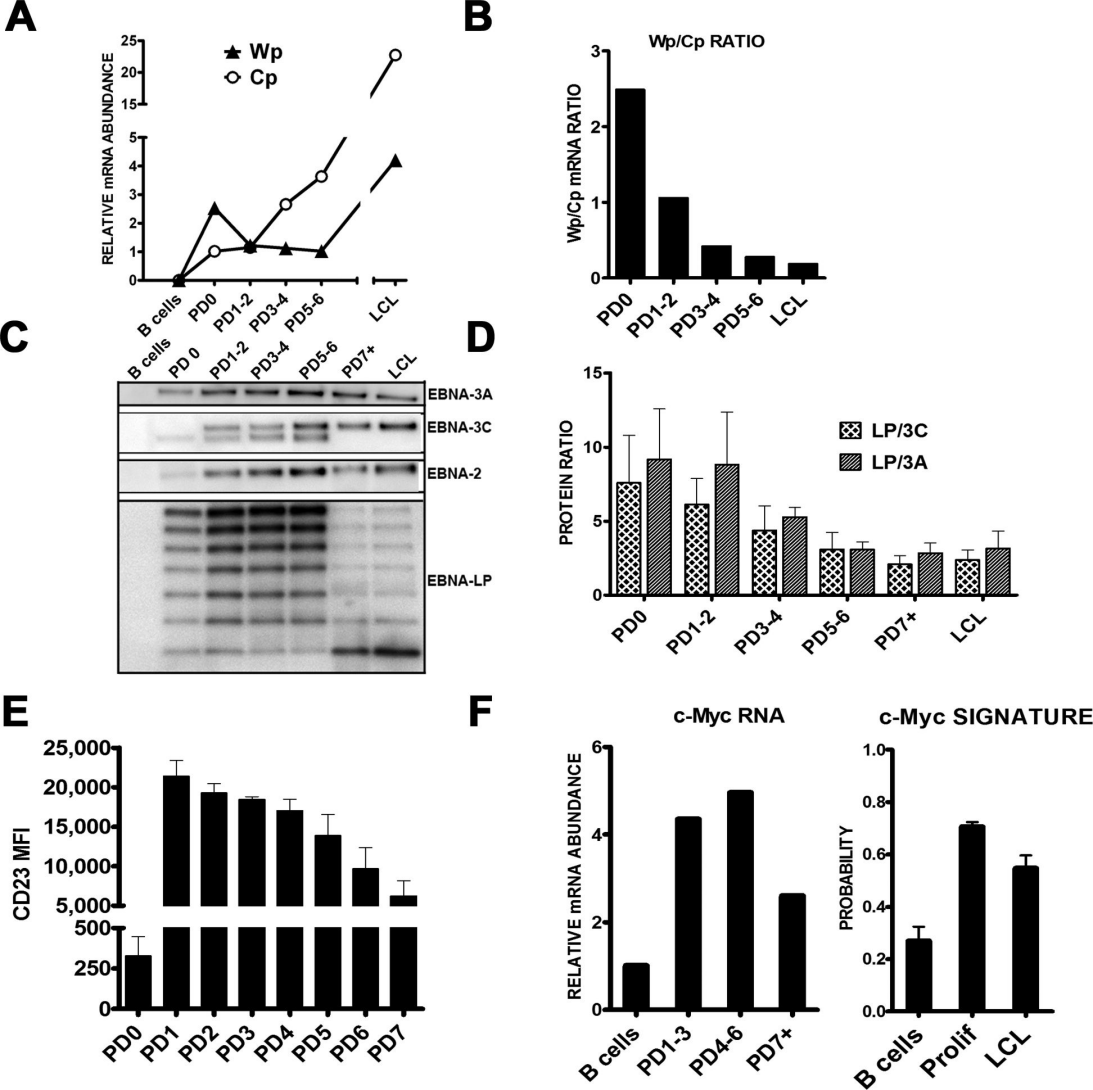
EBV latency and consequential host gene expression changes from initial B cell proliferation through LCL outgrowth (**A**) Expression of Wp (filled triangles) and Cp (open circles) derived mRNAs in EBV-infected cells sorted by population doubling (PD) and monoclonal LCLs. Relative mRNA abundance normalized to a beta-actin control is plotted versus PD. These data are representative of two normal donors and consistent with published time course experiments (Woisetschlaeger et al., 1989; Woisetschlaeger et al., 1990). (**B**) The ratio of Wp to Cp mRNA expression levels from (**A**) is plotted versus division and through LCL outgrowth. (**C**) Protein expression of EBNA-LP, EBNA2, EBNA3A, and EBNA3C are shown from sorted infected PDs and a polyclonal LCL from the same donor. (**D**) Proteins detect by Western blotting from three independent normal donors similar to those in panel (**C**) were quantified. The average ratio of total EBNA-LP protein (i.e., all isoforms) relative to total EBNA3A or EBNA3C +/-SEM is plotted versus PD through LCL. (**E**) Average CD23 surface expression as mean fluorescence intensity (MFI) is plotted versus PD +/-SEM for two donors. (**F, left**) The expression level of c-Myc mRNA is plotted versus sorted PD. (**F, right**) The activity of the c-Myc target gene expression signature (Bild et al., 2006) is plotted from the average expression of targets in microarray samples from four independent donors of resting B cells (B), early proliferating B cells (Prolif), and monoclonal LCLs. Error bars represent SEM.

The dynamic changes in proliferation and DDR associated gene expression support our cell-based assays indicating an early period of ATM/Chk2-mediated growth suppression that is attenuated in later divisions enabling long-term LCL outgrowth. However, to determine whether these changes correlated with viral gene expression, we queried viral transcripts and proteins associated with the latency III growth program in sorted population doublings after infection (Fig. 6 and S5). Wp-associated transcripts were expressed at a markedly higher level than Cp transcripts prior to the first infected cell division (PD0) (Fig. 6A). However, this ratio shifted such that Cp levels were greater after 3-4 cell divisions and through LCL outgrowth consistent with previous observations (Fig. 6B and (Schlager et al., 1996; Woisetschlaeger et al., 1989; Woisetschlaeger et al., 1990)). The consequence of the high Wp/Cp ratio was heightened levels of EBNA-LP protein as well as a heightened EBNA-LP to EBNA3A and 3C protein ratio in early divisions that waned through LCL outgrowth (Fig. 6C-D). Thus, the initial cell divisions characterized by hyper-proliferation display a distinct EBNA gene expression equilibrium that may affect target gene expression.

To more rigorously assess this, we analyzed EBNA2 targets including CD23 (Wang et al., 1991) and c-Myc (Kaiser et al., 1999). In both cases, these EBNA2 targets were highly induced in early cell divisions and then attenuated through LCL outgrowth, still remaining significantly higher than resting B cell levels (Fig. 6E-F). The consequences of the transient increase in c-Myc mRNA was manifested in an increase in the c-Myc target gene expression signature (Bild et al., 2006) during early proliferating cells that was attenuated in LCLs, though still greater than resting B cell levels (Fig. 6F). Given the importance in titrating this potentially genotoxic oncoprotein and the known role of ATM in suppressing c-Myc oncogenesis (Hong et al., 2006; Murphy et al., 2008; Pusapati et al., 2006), these findings strongly support a model of acute oncogenic stress early after EBV infection that is modulated through the well described Wp to Cp switch enabling modest EBNA2 activity critical for indefinite EBV-infected cell outgrowth.

### EBNA3C is required to attenuate the EBV-induced DNA damage response

**Figure 7:**
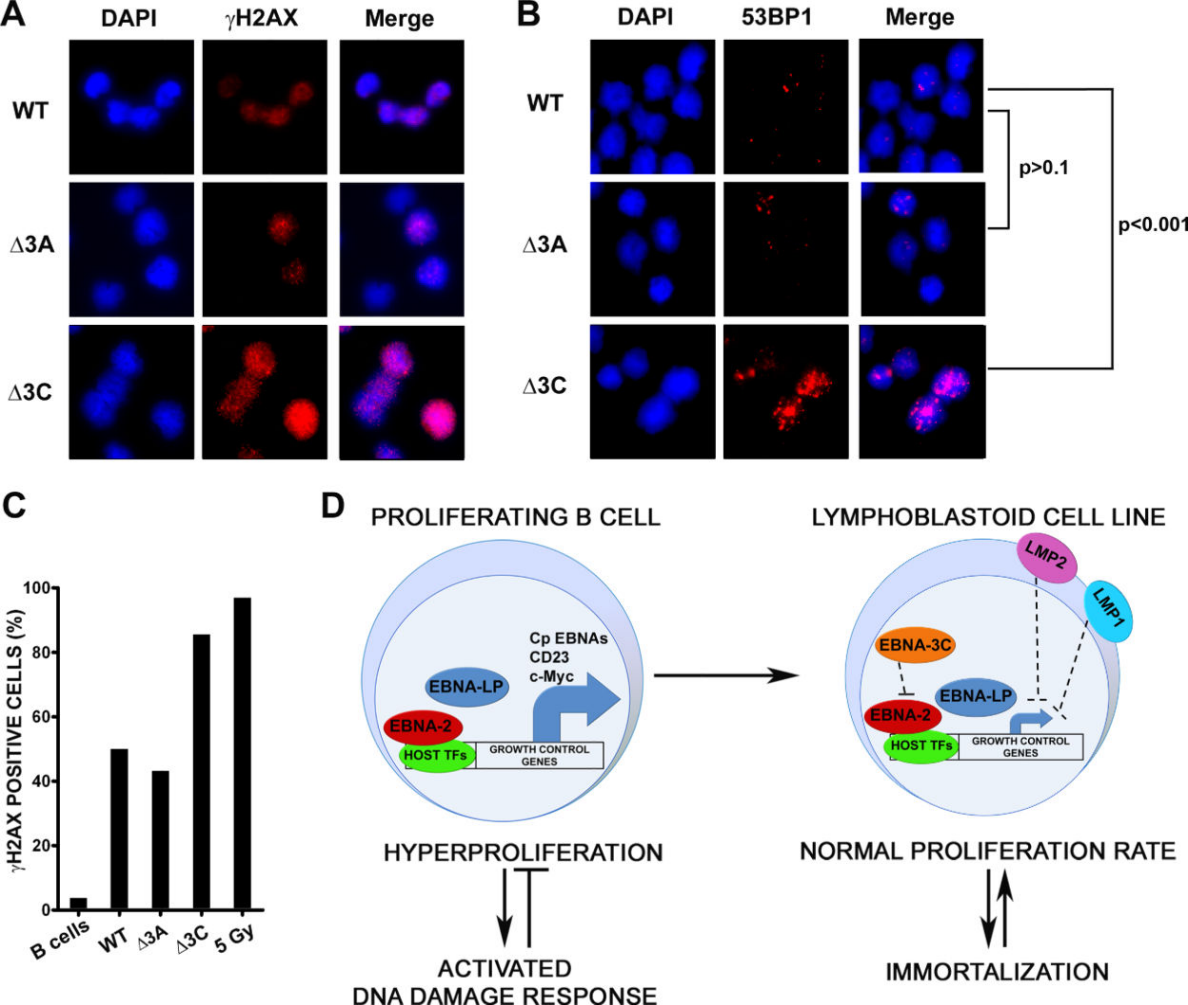
EBNA3C attenuates the EBV-induced DNA damage response (**A**) Representative IF images are shown of γ-H2AX staining (red) from WT, EBNA3A KO (Δ3A), and EBNA3C KO (Δ3C) infected and sorted PD1-4 B cells. DAPI DNA stained (blue) and DAPI/γ-H2AX merged images are also shown. (**B**) Representative IF images are shown of 53BP1 staining (red) from WT, Δ3A, and Δ3C infected and sorted PD1-4 B cells. (**C**) Quantification of IF data from (**A**) is plotted as percentage γ-H2AX positive cells. Average values are plotted for infected cells, uninfected B cells, and 5 Gy γ-irradiated B cells. (**D**) Model for EBV-induced DDR/hyper proliferative period and attenuation during LCL outgrowth. Early in infection EBNA2 and EBNA-LP associate with cellular transcription factors (TF) to potently up-regulate expression of growth control genes and B cell activation markers, including c-Myc and CD23, activating the host DNA damage response (**left**). Later in infection, the activity of the EBNA3 proteins, in particular EBNA3C, down-regulate EBNA2 function, as LMP1 and LMP2 are up-regulated and may cooperate in the constitutive, but attenuated expression of host growth control genes and enhanced cell survival (**right**).

While the induction of the DNA damage response after EBV infection requires latent gene expression and proliferation, a definitive role for viral latent genes in attenuating this response was not demonstrated. In order to determine which latent genes are critical for DDR attenuation during late divisions after infection we chose to interrogate the EBNA3 proteins, EBNA3A and EBNA3C, as they are known to modulate EBNA2 activity. Infection of primary B cells with EBV B95-8, EBNA3A knockout (KO), or EBNA3C KO virus (Anderton et al., 2008) supported early B cell proliferation (Fig. S6A-C). However, upon sorting these early proliferating cells we observed that EBNA3C KO virus infected cells displayed increased activation of the DNA damage response, while EBNA3A KO infected cells were similar to WT B95-8 infection in DDR activation (Fig. 7A-B). Indeed, greater than 80% of EBNA3C KO-infected cells were γ-H2AX positive relative to ~50% of WT or EBNA3A KO-infected cells (Fig. 7C). Similarly, EBNA3C KO-infected cells accumulated 53BP1 DDR foci to a greater extent than WT or EBNA3A KO-infected cells (p<0.001, 3C KO v. WT; p>0.1, 3A KO v. WT). Thus, while B cells infected with either EBNA3A KO or EBNA3C KO virus were crippled for long-term outgrowth (Fig. S6B-C), these experiments define a critical role for EBNA3C in attenuating the host DNA damage response to EBV infection early after infection. These data strongly support our model of a latent gene expression triggered hyper-proliferation induced DDR, followed by proper expression of the EBNA3 proteins, in particular EBNA-3C, in order to attenuate a potentially genotoxic and growth suppressive signaling pathway (Fig. 7D).

## DISCUSSION

It has long been recognized that Epstein-Barr virus transformation efficiency is on the order of 1-10% of infected primary human B cells (Henderson et al., 1977; Sugden and Mark, 1977). However, little is known about the molecular mechanism responsible for this low efficiency. We hypothesize that a robust innate tumor suppressor response is activated by latent viral oncoproteins and blocks outgrowth of the majority of infected cells. Recent evidence suggests that activated oncogene expression is sufficient to trigger a growth-suppressive DNA damage responsive signaling pathway (Halazonetis et al., 2008) and other oncogenic viruses, including the Kaposi's sarcoma-associated herpesvirus, have been shown to induce the DDR after infection or when viral oncoproteins are expressed in primary cells (Dahl et al., 2005; Koopal et al., 2007). Therefore, in this study we asked whether EBV was capable of inducing a DNA damage response in primary B cells and, importantly, whether this response resulted in the low transformation efficiency. We observed that as EBV-infected cells initiated proliferation, a transient DNA damage response (DDR) was activated as evidenced by phosphorylation of ATM Ser1981, H2AX Ser139 (γ-H2AX), Chk2 Thr68, and accumulation of 53BP1 in nuclear foci. Modulation of this signaling pathway by chemical antagonism of ATM and its downstream target Chk2 markedly increased EBV-mediated B cell polyclonal expansion and transformation efficiency thereby demonstrating that the DDR contributes to an EBV-induced innate tumor suppressor pathway. This study identifies a molecular pathway that restricts EBV transformation.

### The source of DNA damage

Towards characterizing the EBV-induced DNA damage signal, we reasoned that either viral or cellular DNA was important for ATM activation. In addition to oncogenic stress, replication intermediates of DNA viruses and retroviruses contain double-stranded DNA ends that activate ATM (Lilley et al., 2007). In fact, EBV lytic replication induces a DDR which is suppressed by inhibition of downstream transcriptional activation of p53 (Kudoh et al., 2005; Mauser et al., 2002). In our studies of primary B cell infection, however, we found no evidence of viral lytic DNA replication or viral DNA associated with DDR activation. First, we did not observe DDR activation within the first 3 days after EBV infection nor did we observe activation using UV-inactivated virus or the non-transforming EBV variant P3HR1 suggesting that the incoming linear DNA genome and tegument proteins within the virion were not responsible for this signal. Second, lytic viral DNA was not responsible for DDR activation as less than 1% of infected cells were undergoing lytic DNA replication when greater than 50% of infected cells were γ-H2AX positive. Third, we asked whether the DNA damage signal was derived from viral episomes since DNA repair factors are recruited to the episome to ensure proper resolution of Holliday junctions following episome replication (Deng et al., 2002; Dheekollu et al., 2007). We observed little increase in episome number per cell and found that viral episomes and γ-H2AX did not co-localize during the period of DDR activation. These data collectively demonstrate that viral DNA is not the source of DNA damage. Our experiments cannot rule out the possibility, however, that viral lytic gene expression downstream of BZLF1 in the absence of lytic DNA replication (Kalla et al., 2010) plays a role in the transient DDR early after infection. Despite this possibility, we inferred from our data that viral latent gene expression causes an oncogenic stress response leading to cellular DNA damage.

The initiation of cell proliferation defines the period after EBV infection when ATM and Chk2 were active in suppressing transformation. Rigorous analysis of infected cell division rates uncovered a period of hyper-proliferation where early population doublings (PDs) were every 8-12h leading to DDR activation, while later divisions displayed an attenuated rate of ~24-30h per division similar to LCLs and had little evidence of DDR activation. Microarray analysis of gene expression during the transition from resting B cell to early EBV-induced hyper-proliferation and through LCL outgrowth strongly supported our cell-based observations. Specifically, genes involved in proliferation and the DDR, including ATM/p53-dependent targets (Elkon et al., 2005), were highly induced early after infection and then attenuated during the transition to LCL. We propose that aberrant induction of cellular DNA replication early after EBV infection activates a DNA damage response that is dependent on EBNA2 and EBNA-LP mediated up-regulation of S phase promoting oncoproteins including c-Myc, cyclin D2, and E2F1 ((Kaiser et al., 1999; Sinclair et al., 1994) and Fig. 6). Indeed, we observed increased expression of c-Myc and its gene activation signature in hyper-proliferating cells relative to LCLs. Furthermore, EBNA-LP protein levels and Wp-derived transcripts were heightened during this early period relative to EBNA3 proteins and Cp transcripts consistent with previous analysis of the initial cascade of viral latent gene expression at different days post infection (Schlager et al., 1996; Woisetschlaeger et al., 1989; Woisetschlaeger et al., 1990). Finally, EBNA3C, but not EBNA3A, deleted virus-infected cells displayed a significantly stronger DDR during early proliferation. Thus, while both EBNA3A and EBNA3C likely mitigate growth arrest in LCLs through p16 suppression (Hertle et al., 2009; Skalska et al., 2010), during early outgrowth EBNA3C is also required to modulate the DNA damage response. Collectively, our data support a model that initial EBV-driven hyper-proliferation leads to an oncogenic stress that is ultimately attenuated as EBNA3 proteins moderate EBNA2 driven c-Myc and its genotoxic and growth suppressive consequences. This ultimate balance in viral and host gene expression enables constitutive S phase induction without driving selection of cells with genomic instability.

### *In vivo* implications

Our findings have implications pertaining to the germinal center model for EBV infection (Roughan and Thorley-Lawson, 2009) in the context of B cell lymphomagenesis. In particular, our observed hyper-proliferative phase early after infection *in vitro* may be similarly induced by EBV *in vivo* and is reminiscent of B cell proliferation rates in the germinal center (MacLennan, 1994). Bcl-6 down-regulation of the DDR mitigates the consequences of centroblast hyper-proliferation in the germinal center (Ranuncolo et al., 2007), while EBV potently suppresses Bcl-6 early after infection leaving DDR checkpoints intact (Siemer et al., 2008). *In vivo*, an EBV-induced hyper-proliferative period after primary infection may promote extra-follicular B cell maturation or drive EBV-infected naïve B cells into GCs. However, a critical balance must be struck between the aberrant latent oncoprotein-driven proliferation early after infection and the stable proliferative signals found in LCLs to maintain an activated, immortalized state. Perturbations in this balance *in vivo* may select for mutations driving lymphomagenesis. For example, the *IgH/c-myc* translocation common in Burkitt's lymphoma (BL) may be the consequence of such an event. Given our findings, it is plausible that imbalances in EBV latent gene expression may provide a milieu of cells with an increased potential for genomic instability. Recent work in BL cell lines suggests that this is likely the case.

EBV infection of BL cell lines or heterologous expression of EBNA1, EBNA3C, or LMP1 in BL cell lines increased the frequency of non-clonal chromosomal aberrations (Gruhne et al., 2009a; Gruhne et al., 2009b). EBNA1 increased reactive oxygen species (ROS) through transcriptional up-regulation of NOX2, EBNA3C perturbed mitotic spindle checkpoints through BubR1 down-regulation, and LMP1 attenuated ATM protein levels and decreased DNA repair. EBNA3C has also been shown to modulate the activity of Chk2 (Choudhuri et al., 2007). However, these three viral proteins are constitutively expressed in LCLs in the absence of overt genomic aberrations. Therefore, we expect that these findings unmask activities that may link the aforementioned potential for imbalanced gene expression to tumorigenesis. In our system, we did not observe increased transformation in the presence of anti-oxidants including N-acetyl cysteine and citric acid (data not shown) suggesting that EBNA1 induced ROS was not responsible for the EBV-induced DDR. We also did not observe changes in BubR1 or ATM expression through LCL outgrowth (data not shown). However, we anticipate that genomic instability may ensue in the setting of aberrant latent oncoprotein expression that may exist in BL and other EBV-associated tumors. Consistent with this notion and our findings, a recent report suggests that while LCLs maintain a stable karyotype, early DNA damaging events may lead to non-clonal chromosomal aberrations including telomere fusions (Lacoste et al., 2009). This report supports our findings of an early hyper-proliferation associated oncogenic stress that may induce such structures leading to ATM activation (Karlseder et al., 1999) and suppression of long-term outgrowth. Thus, only cells with the ability to maintain a stable karyotype emerge as LCLs.

### Summary

Our study provides the characterization of an innate tumor suppressor pathway that regulates EBV immortalization of B cells. This pathway depends on ATM and Chk2, which are activated early after infection during a period of hyper-proliferation. The initial high level expression of EBNA2 and EBNA-LP target genes such as c-Myc leads to DDR activation. Following this initial period, the activity of additional viral latent proteins, including EBNA3C and possibly LMP1 and LMP2, is important for attenuating early gene expression targets, limiting activation of the DDR, and ensuring cell survival. The end result of these dynamic changes in viral and cellular gene expression is outgrowth of a constitutively activated lymphoblastoid cell line harboring a stable karyotype. However, perturbations in this gene expression program through loss of upstream control by viral latent proteins may lead to the progression of EBV-associated lymphomas. Our studies provide a model for the study of EBV transformation accounting for dynamic viral and host changes during the early period following primary B cell infection.

## Supplementary Material

01

## EXPERIMENTAL PROCEDURES

### Antibodies

Primary antibodies to γH2AX, pATM Ser1981, and 53BP1 (Cell Signaling #2577, #4526, and #4937) were used for IF at 1:50. Alexa488 goat α-mouse and Alexa 568 goat α-rabbit were used as secondary antibodies (Molecular Probes #A-11029 and #A-11011). Rabbit α-human Chk2 or pChk2 Thr68 (Cell Signaling #2662 or #2661) and mouse α- EBNA-LP (JF-186, 1:250), EBNA2 (PE2, 1:100), EBNA3A (Exalpha #F115P, 1:500), and EBNA3C (A10, gift of E. Johannsen 1:2500) were used for Western blotting.

### Fluorescence microscopy

#### Immunofluorescence (IF)

3 × 10^5^ B cells were pelleted, washed in PBS, resuspended in 25 μl of PBS, spread on a microscope slide and dried at 37°C for 20 minutes then fixed in 4% PFA in PBS for 15 minutes, permeabilized in PBS containing 0.5% Tween-20 for 20 minutes then blocked in PBS with 0.2% Tween-20 containing 5% normal goat serum for 1 hour. Indirect IF was performed as described in (Bridger and Lichter, 1999). Slides were mounted in Vectashield containing DAPI (Vector Laboratories).

#### Fluorescence in situ hybridization (FISH)

Cells were fixed in methanol acetic acid as described previously (Sullivan and Warburton, 1999). The EBV genome containing bacterial artificial chromosome MD-1 (kindly provided by F. Wang, Harvard Medical School) was labeled with fluorescent green-dUTP using a nick translation kit (Abbott Molecular) following manufacturer's instructions. Slides were denatured in 70% formamide, hybridized and washed as described previously (Sullivan and Warburton, 1999) except with using fluorescent probes, slides were not blocked then incubated with a fluorescent secondary antibody.

#### IF/FISH

Slides were fixed for IF in 2% paraformaldehyde in PBS. Antibodies were added to slides and denatured as previously described (Sullivan and Warburton, 1999) except that slides were denatured for 6 minutes. Slides were hybridized and washed as described above.

Data analysis was performed as described in Supplemental Experimental Procedures.

### Flow cytometry analysis

#### B cell proliferation assays

6-carboxyfluorescein succinimidyl ester (CFSE, Sigma, # 21888)-stained or CellTrace Violet (Invitrogen, #C34557)-stained PBMC were infected with EBV and incubated with different concentrations of ATMi, Chk2i, or DMSO during different periods. Proliferation of CD19^+^ cells was assayed by flow cytometry as a ratio of CFSE^low^ cells to total PBMC at 14 days after infection. Detailed kinetics of EBV proliferation was determined as described in Fig. S3 (Hawkins et al., 2007).
