# Supplementary material for: An ATM/Chk2-mediated DNA damage responsive signaling pathway suppresses Epstein-Barr virus transformation of primary human B cells
Source: Cell Host Microbe. Author manuscript; Available in PMC 2011 Dec 16. (PMC3049316; doi:10.1016/j.chom.2010.11.004)
Supplement: 01 [file NIHMS256435-supplement-01.doc]

**
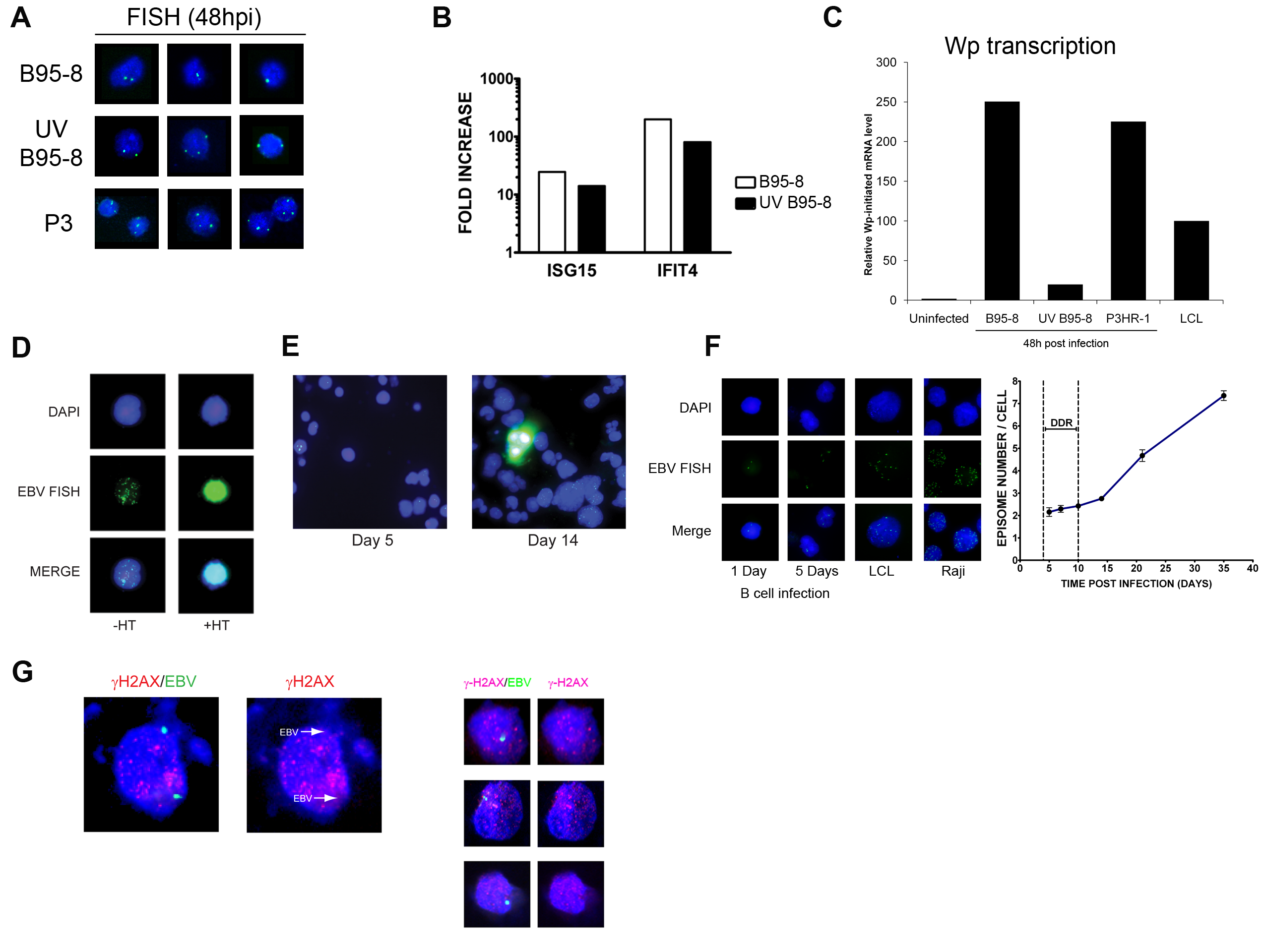
**

**Supplemental Figure 1, related to Figure 1.** **Nuclear genome deposition and the analysis of viral and host gene expression following primary B cell infection with EBV B95-8, UV-inactivated B95-8, P3-HR1 (A-C).** **EBV genomes in infected primary B cells are independent of γ-H2AX foci (D-G).** (**A**) Fluorescence *in situ* hybridization (FISH) of viral genomes 2 days post B cell infection with EBV B95-8, UV-inactivated B95-8 (UV B95-8), and P3HR1 (P3) at an MOI of ~5. (**B**) Quantitative RT-PCR of the interferon responsive mRNAs ISG15 and IFIT4 24h following PBMC infection with B95-8 and UV-inactivated B95-8. mRNA levels were normalized to GAPDH and primers were as described previously (Martin et al., 2007) (**C**) Quantitative RT-PCR for Wp-initiated mRNAs 48h after PBMC infection with B95-8, UV-B95-8, or P3 virus strains. Analysis of the relative levels of W0 – W1/W2 containing mRNAs indicate that infection was equivalent between B95-8 and P3, while UV-inactivated virus was essentially unable to produce Wp-initiated transcripts. mRNAs were normalized to GAPDH and primers for these experiments were previously described (Bell et al., 2006). (D) Fluorescence *in situ* hybridization (FISH) of EBV genomes (green) in B95-8 Z-HT cells (Johannsen et al., 2004). Uninduced cells (-HT) mostly contained latent episomes (**left**). A representative cell undergoing lytic replication following exposure to 4-hydroxytamoxifen (+HT) is shown on the **right**. **(E)** EBV FISH in B cells 5 days and 14 days after infection. Intense lytic staining was rarely observed 5 days after infection (**left**). Approximately 1-5% of cells were undergoing lytic DNA replication by FISH at 14 days (**right**). **(F)** **Left**, Representative images of EBV genomic FISH in primary CD19+ B cells at 1 and 5 days post infection with B95-8 (MOI ~5), steady state 8-10 genomes in a LCL, and 50 genomes per Raji cell. **Right**, Episome number as determined by FISH at different times after infection. The y-axis represents the average number of episomes per cell, the x-axis represents days after infection when cells were collected and fixed for hybridization (5, 7, 10, 14, 21, and 35 days). Vertical dashed lines show the beginning and end of the period in which the DNA damage response (DDR) was observed. Error bars represent SEM. These data were collected from greater than 50 nuclei at each time point for three independent normal donors. **(G)** **Left**, IF/FISH of γH2AX (red) and EBV genomes (green) in B cells 7 days post infection. Left cell shows IF/FISH, right cell shows only IF for γH2AX with white arrows representing EBV genome location. The **right** panels are additional representative IF/FISH and IF images.


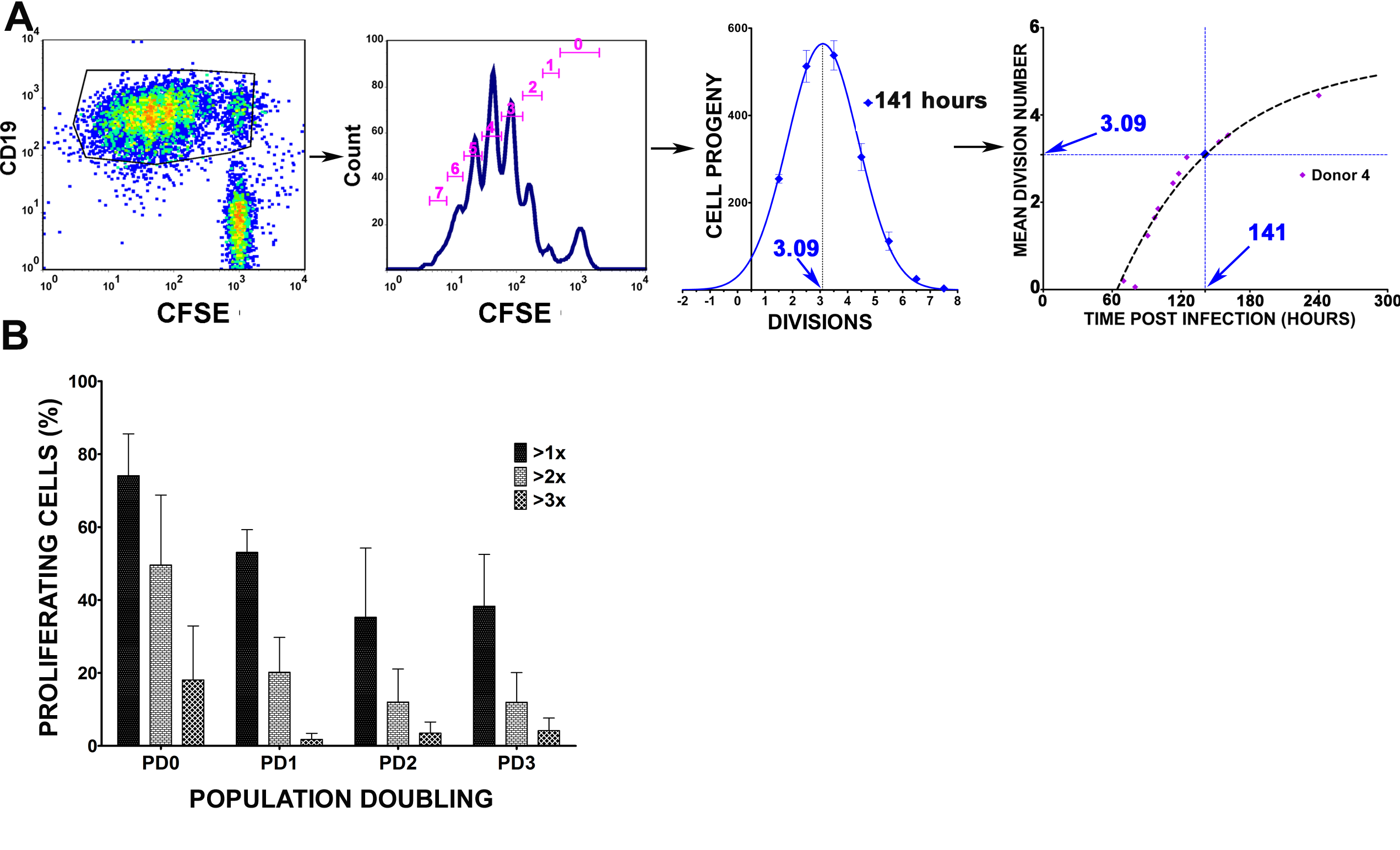


**Supplemental Figure 2, related to Figure 2. CFSE-based kinetic analysis of EBV-induced proliferation. (A)** Analysis of EBV-infected PBMC stained by CFSE was performed by FACS at different time of infection (141 hour post infection point is shown, left). CFSE-based population doublings of CD19+ B cells were determined using the “Proliferation” tool in the FlowJo program. Number of cells in each PD (N) was normalized to averaged division number i (i=i+0.5) and fit to a Gaussian distribution for each time point. Mean division number was determined as shown (MDN=3.09 for 141 hour p.i. for a given donor, middle). Then, MDN over time post infection plot was generated for each time point (right). **(B)** CD19+ cells from population doublings 0, 1, 2 and 3 were double-sorted by flow-cytometry and incubated separately for 48 hours. Then, proliferative potency of cells from each population doubling was measured by FACS as a percent of cells divided more than once, twice or thrice. The data shown is the average for 2 donors. Error bars represent standard error.


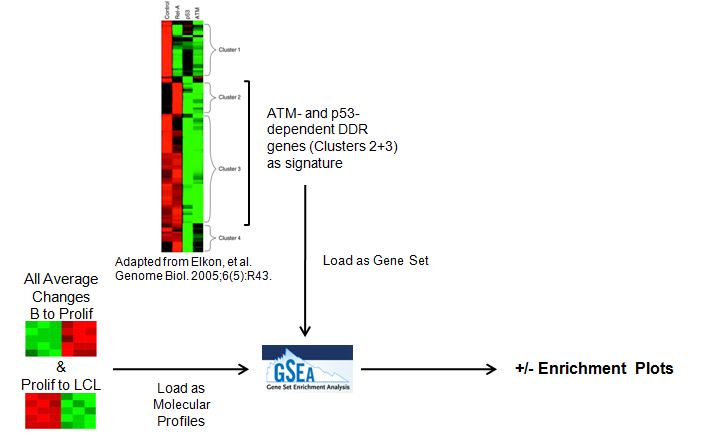


**Supplemental Figure 3, related to Figure 3. Schematic diagram of GSEA analysis performed on B cells, Proliferating cells, and LCL microarray data with ATM/p53 target genes.** We imported our average mRNA expression changes from either B to Proliferating (Prolif) or Prolif to LCL as molecular profiles. Then we assigned the ATM and p53 target genes as identified from (Elkon et al., 2005) as a gene set into GSEA. The enrichment of this gene set relative to 1000 random permutations within the B to Prolif samples was plotted in the data in Fig. 4C, which indicated that EBV-induced proliferation was associated with an activation of the ATM/p53 gene expression signature, while from proliferation through LCL outgrowth this gene set was depleted indicating attenuation of ATM/p53 target gene expression.

**
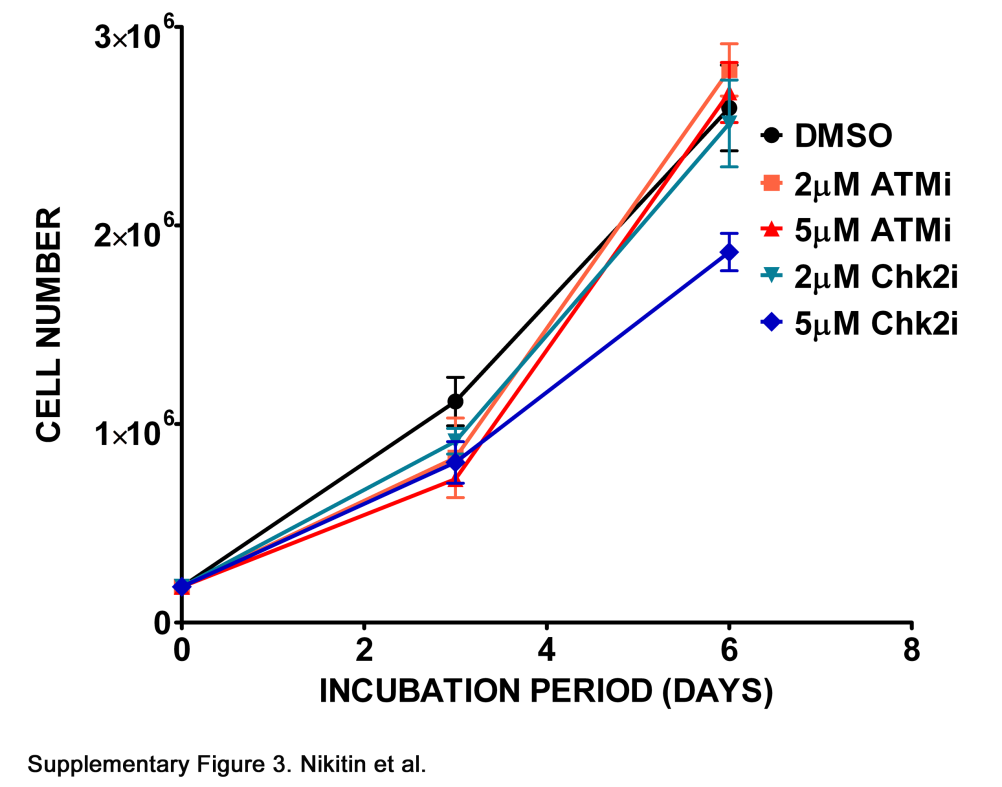
**

**Supplemental Figure 4, related to Figure 5. ATM and Chk2 inhibition did not increase the growth of LCLs.** Three recently-derived LCL lines were incubated at 2 x 10^5^ cells/mL with DMSO, 2 μM ATMi, 5 μM ATMi, 2 μM Chk2i or 5 μM Chk2i for 6 days. Cell number was counted microscopically using the Trypan Blue exclusion method at days 3 and 6 and plotted against time. The results shown are the average of the three independent lines with error bars representing standard error of the mean.


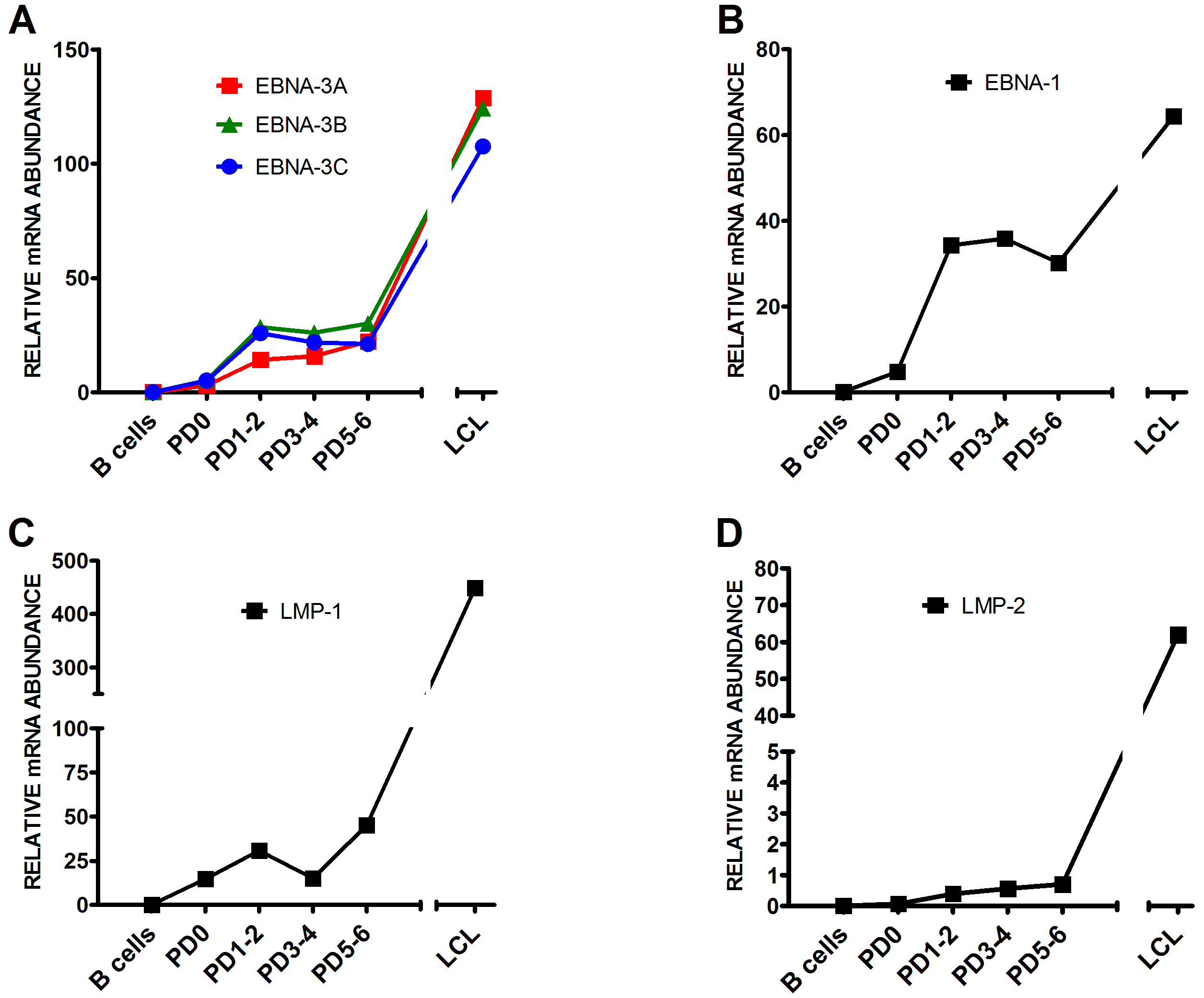
**Supplemental Figure 5, related to Figure 6. Expression of viral latency genes during early cell divisions through LCL outgrowth.** The expression of (**A**) EBNA-3A, EBNA-3B, and EBNA-3C, (**B**) EBNA-1, (**C**) LMP1, and (**D**) LMP2A was detected by quantitative real-time PCR. EBV-infected PBMC were sorted 5 and 7 days post infection by CD19 and CFSE for population doublings and also a polyclonal LCL was derived from the same infected donor (day 35 post infection). RNA was extracted and SYBR green-based qRT-PCR was performed using primers spanning exons as previously described (EBNA1 Y3/U/K, LMP1 exon 2/3, and LMP2A exon 1/2 from (Bell et al., 2006) and EBNA-3A (exon1/2), EBNA-3B (exon 1/2), and EBNA-3C (exon 1/2) from (Sengupta et al., 2006).Data from one normal donor are shown in the graphs which are representative of three independent infections. The relative mRNA abundance is plotted normalized to an HPRT mRNA control (primers from (Ranuncolo et al., 2007)). We note that for each mRNA, an increase is observed from PD5-6 through LCL outgrowth, which is likely due to an increase in genome copy number.

**Supplemental Figure 6, related to Figure 7. Characterization of EBNA-3A KO and EBNA-3C KO viruses. (A)** The protein expression of EBNA-3A, -3C, -2, -LP and LMP-1 was analyzed 10 days after B cell infection with EBV by western blotting. EBV-negative BJAB and EBV positive EF-3D LCL were used as controls. (**B**) (Top) Dot plot analysis of EBV-driven B cell proliferation is depicted using the CD19 B-cell marker (y-axis) and CellTrace Violet dye (x-axis). Both EBNA-3A and 3C KO viruses drive B cell proliferation at 7 days post infection. (Bottom) Similar dot plots of CD19/Violet indicate that EBNA-3A and EBNA-3C KO viruses are impaired in driving B cell proliferation through 14 days after infection compare to WT virus. (**C**) Quantification of proliferating CD19^+^ B cells, infected with WT, EBNA-3A KO and EBNA-3C KO EBV at day 7 and day 14 post infection. Average for two normal donors is shown +/- SEM.

**SUPPLEMENTAL EXPERIMENTAL PROCEDURES**

**Viruses, Cells and Reagents**. B95-8 virus was produced from the B95-8 Z-HT cell line as previously described (Johannsen et al., 2004). P3HR1 virus was generated from P3HR1 Z-HT cells (kindly provided by Ellen Cahir-McFarland (Harvard Medical School)). Buffy coats were obtained from normal donors through the Carolina Red Cross and peripheral blood mononuclear cells (PBMC) were isolated by Ficoll Hystopaque gradient (Sigma #H8889). CD19+ B cells were either purified from PBMC using the BD iMag Negative Isolation Kit (BD, cat #558007) or from buffy coats using the RosetteSep kit (Stem Cell #15024). Purity was routinely greater than 90% as determined by flow cytometry. Primary cells were cultured in RPMI 1640 plus 15% fetal calf serum (FCS), 2 mM L-glutamine, and penicillin/streptomycin (R15), while LCLs were cultured in similar media containing only 10% FCS (R10).

*Kinases inhibitors*. ATM was inhibited using KU-55933, or ATMi (Hickson et al., 2004) (EMD #118500). Chk2 was inhibited using the 2-arylbenzamidazole compound Chk2i II (Arienti et al., 2005) (EMD #220486).

*Cell infection*. All bulk infections were performed by incubating cells with B95-8 Z-HT supernatants or GFP expression construct containing B95-8 (WT), EBNA-3A KO B95-8 or EBNA-3C KO concentrated supernatant for 1h at 37°C in a CO_2_ incubator followed by washing twice in PBS and resuspending cells in R15 media. For IF studies, each 10^6^ primary CD19+ B cells were infected with 500 μL of B95-8 Z-HT supernatant, or 10^7^ PBMC were infected with 100,000 Green Raji Units of WT, EBNA-3A KO or EBNA-3C KO B95-8 EBV and sorted for CD19+ proliferating B cells at 7 days post infection. For proliferation assays, 500 μL of B95-8 Z-HT supernatant virus was used per 10^7^ PBMC (~10^6^ CD19+ cells).

**Antibodies**. Monoclonal antibodies to EBNA-LP were purified from the supernatant of the JF-186 hybridoma cell line (Finke et al., 1987). Mouse anti-human CD19 antibodies conjugated with either APC (BD Bioscience, #555415) or PE (BD, #555413) were used as surface B cell markers in flow cytometry.

**Transformation assays.** Infection of human PBMC by B95-8 EBV was performed in the presence of 0.1% DMSO, 2 μM or 5 μM ATMi or Chk2i added at different times post infection. B95-8 Z-HT supernatant was titrated from 1 ml/10^7^ PBMC to 30 μL/10^7^ PBMC. 2.5 x 10^6^ infected PBMC were seeded in 24 wells of a 96-well plate for each infection point. The percentages of wells positive for B cell outgrowth (LCL) at five weeks post infection were plotted relative to the amount of virus used per well. The efficiency of transformation was determined as published (Henderson et al., 1977) where the amount of B95-8 virus necessary to yield 62.5% of positive wells was considered 1 transforming unit (TU) per well.

**Flow cytometry analysis.** *B cell proliferation assay.* A secondary proliferation assay was done using the dye PKH26 (Sigma, #MINI-26) similar to that described above. However, upon sorting CD19+ B infected cells into early (PD1-4) and late (PD6+) divisions, cells were secondarily stained with CFSE and followed daily for proliferation (CFSE^low^ cells) and viability. A population of cells with low FSC signal, which was also found to be largely 7-AAD positive (data not shown), was measured as a proxy for loss of cell viability.

*Cell Sorting*. CD19^+^ B cells were sorted for population doublings PD0-PD7 based on CFSE intensity at different times after EBV infection on FACS Diva, MoFlo or FACSVantage sorters using two, three or four way sorts. Sorted populations of B cells were either examined by IF/FISH, Western blot, or microarray analysis or returned to culture for further analysis.

**Fluorescence microscopy data analysis.** Images were captured using a Nikon Eclipse TS100 microscope with a 60X Nikon objective and a Photometrix Cool Snap camera, using the Nikon F imaging software provided. All IF images were taken using the same capture settings between samples. Images were pseudo-colored in NIS Basic Research (BR, Nikon). Quantification of the fluorescence intensity was performed in BR by identifying each nucleus as a region of interest (ROI) in a field using the DAPI image. The ROIs were then overlaid on the fluorescein and rhodamine images from the same field where mean pixel intensity was assigned to each ROI and exported to MS Excel for additional analysis. For episome number analysis in BR, ROIs were assigned as before. The ROIs would then be overlaid on the FISH image of the same field. Using the object count option, the number of fluorescent objects in each region of interest was assigned and counts were exported to MS Excel for further analysis.

**Gene expression analysis**. *Quantitative real-time RT-PCR.* RNA was extracted using QIAgen RNeasy from purified primary B cells, infected cells sorted using CFSE on cell division, and LCLs derived from the same donor. Reverse transcription followed by random cDNA priming was as described (Forte and Luftig, 2009). Real-time PCR was performed either using Quanta SYBR Green or specific Taqman-based probes in an Applied Biosystems Step One Plus instrument. Wp- and Cp-derived transcripts were detected using primers flanking the W0-W1/W2 and C0-W1/W2 boundaries, respectively, and a probe from within W1 as described (Bell et al., 2006). EBNA1, 3A, 3B, 3C and LMP1, and 2A mRNAs were detected by SYBR green with primers described in the supplementary information.

*Western blotting*. Samples were lysed in a 1% TritonX-100 containing buffer (20 mM Tris, pH 7.5, 100 mM NaCl, 10% glycerol, 1% Triton X-100, 1 mM EDTA, 1 mM DTT, 20 mM NaF, 10 mM sodium pyrophosphate, and Complete protease inhibitors without EDTA) and normalized to total protein content by BCA assay. All samples were run on Novex 4-12% Bis-Tris gels and blotted using standard procedures. Antibodies used were described above.

*Microarray analysis and gene enrichment.* Total mRNA was prepared from four normal donors in three conditions: i) uninfected purified CD19^+^ B cells, ii) infected PBMC, sorted at 6 days post infection for PD1-PD4, iii) monoclonal LCL derived by limiting virus dilution on PBMC. cDNA preparation, labeling, and fragmentation was performed using the Gene Chip wt cDNA synthesis and amplification kit (Affymetrix # 900673) and Exon Array labeling kits (Affymetrix # 900671). 12 samples (4 of each condition) were hybridized to HuEx 1.0ST Exon Arrays (Affymetrix # 900650) and the chips were scanned in the Duke Microarray Facility. The resultant CEL files were RMA normalized and the data was analyzed with GenePattern (Reich et al., 2006) and GSEA v2 (Subramanian et al., 2005) to analyze the ATM/p53 target data set (Elkon et al., 2005). Differential expression of genes was evaluated by applying 2-way ANOVA or SAM (Tusher et al., 2001). Gene ontology categories were assessed using the GATHER algorithm (Chang and Nevins, 2006).

*Myc signature analysis.* Affymetrix HuEx Exon array data was RMA normalized using Expression Console software obtained from Affymetrix website. To enable the use of Myc pathway signature generated on the U133A chip (Bild et al., 2006), a bridging file was generated by aligning U133 plus 2 chip probeset target sequences to Mar 2006 genome and comparing their genomic coordinates with the Affymetrix provided coordinates of the HuEx probesets. The resulting bridging file was used to merge the two datasets by using FileMerger ([http://www.filemerger.genome.duke.edu](http://www.filemerger.genome.duke.edu/)). The training data, as represented by Myc or GFP was designated 1 or 0, respectively, while the validation data was labeled 2 to assess the probability of the individual samples with the Myc pathway activation using Bayesian binary regression analysis (BINREG) in MATLAB. The details of the parameters used to assess the Myc pathway probability (i.e. gene numbers, metagenes) as well as the validation and optimization strategy is described elsewhere (Gatza et al., 2010).

**SUPPLEMENTAL REFERENCES**

Bell, A.I., Groves, K., Kelly, G.L., Croom-Carter, D., Hui, E., Chan, A.T., and Rickinson, A.B. (2006). Analysis of Epstein-Barr virus latent gene expression in endemic Burkitt's lymphoma and nasopharyngeal carcinoma tumour cells by using quantitative real-time PCR assays. J Gen Virol *87*, 2885-2890.

Elkon, R., Rashi-Elkeles, S., Lerenthal, Y., Linhart, C., Tenne, T., Amariglio, N., Rechavi, G., Shamir, R., and Shiloh, Y. (2005). Dissection of a DNA-damage-induced transcriptional network using a combination of microarrays, RNA interference and computational promoter analysis. Genome Biol *6*, R43.

Finke, J., Rowe, M., Kallin, B., Ernberg, I., Rosen, A., Dillner, J., and Klein, G. (1987). Monoclonal and polyclonal antibodies against Epstein-Barr virus nuclear antigen 5 (EBNA-5) detect multiple protein species in Burkitt's lymphoma and lymphoblastoid cell lines. J Virol *61*, 3870-3878.

Martin, H.J., Lee, J.M., Walls, D., and Hayward, S.D. (2007). Manipulation of the toll-like receptor 7 signaling pathway by Epstein-Barr virus. J Virol *81*, 9748-9758.

Ranuncolo, S.M., Polo, J.M., Dierov, J., Singer, M., Kuo, T., Greally, J., Green, R., Carroll, M., and Melnick, A. (2007). Bcl-6 mediates the germinal center B cell phenotype and lymphomagenesis through transcriptional repression of the DNA-damage sensor ATR. Nat Immunol *8*, 705-714.

Sengupta, S., den Boon, J.A., Chen, I.H., Newton, M.A., Dahl, D.B., Chen, M., Cheng, Y.J., Westra, W.H., Chen, C.J., Hildesheim, A.*, et al.* (2006). Genome-wide expression profiling reveals EBV-associated inhibition of MHC class I expression in nasopharyngeal carcinoma. Cancer Res *66*, 7999-8006.
